# P-1221. In Vitro Activity of Cefiderocol Against Clinical Strains Collected from China in 2023

**DOI:** 10.1093/ofid/ofaf695.1414

**Published:** 2026-01-11

**Authors:** Dandan Yin, yan Guo, Naoki Kohira, Xin Zhao, Fupin Hu

**Affiliations:** Huashan Hospital, shanghai, Shanghai, China (People's Republic); Huashan Hospital, shanghai, Shanghai, China (People's Republic); Shionogi & Co., Ltd., Toyonaka, Osaka, Japan; Shionogi China Co., Ltd., shanghai, Shanghai, China; Huashan Hospital, shanghai, Shanghai, China (People's Republic)

## Abstract

**Background:**

Cefiderocol, a siderophore cephalosporin, combats Gram-negative pathogens by hijacking iron transport to accumulate in the periplasm and inhibit PBP3-mediated cell wall synthesis. Here we evaluated the in vitro activity of cefiderocol against Gram-negative bacilli isolated from clinical settings in China in 2023, with a focus on meropenem-resistant isolates.

**Figure:**
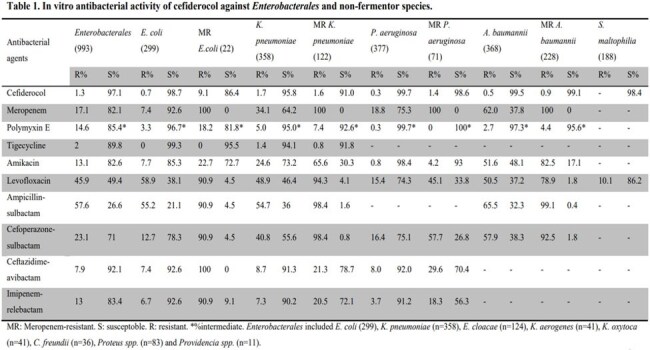

**Methods:**

A total of 2,049 clinical strains, were collected from 63 hospitals across China, including *Enterobacterales* (n=993), *Pseudomonas aeruginosa* (n=377), *Acinetobacter baumannii* (n=368), *Stenotrophomonas maltophilia* (n=188), and *Burkholderia cepacia* (n=123). Antimicrobial susceptibility testing was performed using the broth microdilution according to CLSI M100 (2024) standards, with iron-depleted cation-adjusted Mueller-Hinton broth for cefiderocol. Minimum inhibitory concentrations (MICs) were interpreted according to CLSI breakpoints.

**Results:**

About 50.8% (1040/2049), 15.8% (323/2049) and 9.3% (190/2049) of the isolates were obtained from respiratory tract, urinary tract and blood, respectively. The MIC_50_ values of cefiderocol against *Enterobacterales* ranged from ≤ 0.03 mg/L to 0.25 mg/L, MIC_90_ values ranged from 0.25 mg/L to 4 mg/L, and susceptibilities ranged from 93.5% to 100% (Table 1; data shown for *Enterobacterales, E. coli* and *K. pneumoniae*). For meropenem-resistant *E. coli* and *K. pneumoniae*, the MIC_50_ values were both 2 mg/L, and MIC_90_ values were 8 mg/L and 4 mg/L, with susceptibilities of 86.4% and 91.0%, respectively. Susceptibility for *P. aeruginosa, A. baumannii*, including meropenem-resistant isolates, and *S. maltophilia* ranged from 98.4% to 99.7% (Table 1). For *B. cepacia*, MIC_50_ and MIC_90_ values were 0.06 mg/L and 0.25 mg/L, respectively. Cefiderocol showed similar antibacterial activity with polymyxin E and tigecycline against most bacteria, outperforming carbapenems and β-lactam/β-lactamase inhibitor combinations. In particular against meropenem-resistant strains, cefiderocol was one of the most active agents, except for tigecyline or polymyxin E.

**Conclusion:**

Cefiderocol exhibits significant potential as a critical therapeutic option for Gram-negative bacterial infections in China.

**Disclosures:**

Xin Zhao, n/a, Shionogi China Co., Ltd.: Grant/Research Support

